# A Child with Prostaglandin I_2_-associated Thyrotoxicosis: Case Report

**DOI:** 10.4274/jcrpe.galenos.2018.2018.0169

**Published:** 2019-05-28

**Authors:** Yuri Sonoda, Kenichiro Yamamura, Kanako Ishii, Kazuhiro Ohkubo, Kenji Ihara, Yasunari Sakai, Shouichi Ohga

**Affiliations:** 1 Kyushu University Graduate School of Medical Sciences, Department of Pediatrics, Fukuoka, Japan; 2 Oita University Faculty of Medicine, Department of Pediatrics, Oita, Japan

**Keywords:** Prostaglandin I2, pulmonary arterial hypertension, congenital portosystemic venous shunt syndrome, hyperthyroidism

## Abstract

Prostaglandin I_2_ (PGI_2_) causes hyperthyroidism, a critical complication in patients with pulmonary arterial hypertension (PAH). However, it remains unknown whether PGI_2_ may have unfavorable effects on thyroid function in children with congenital portosystemic venous shunt syndrome (CPSVS). We present a boy with CPSVS who developed PAH at seven years of age. During ongoing PGI_2_ therapy, he experienced thyrotoxicosis at 17 years of age. The literature review showed that the reported 12 patients with PAH (median 11 years of age) developed hyperthyroidism during between one and 11 years of PGI_2_ treatment. Only one patient survived the acute PAH crisis due to hyperthyroidism. These data provide evidence that prophylactic intervention for hyperthyroidism is indicated for children with CPSVS during PGI_2_ treatment.

**What is already known on this topic?**

Continuous intravenous injection of epoprostenol prostaglandin I_2_ (PGI_2_) is an effective medication for patients with severe cardiac failure due to pulmonary artery hypertension. PGI_2_ may cause the life-threatening side effect of hyperthyroidism with an incidence rate of 6.7%.

**What this study adds?**

We report the first pediatric case with portosystemic venous shunt syndrome, a patient who developed thyrotoxicosis after 10 years of prostaglandin I_2_ (PGI_2_) treatment. Prophylactic monitoring of thyroid function is mandatory for pediatric pulmonary artery hypertension patients undergoing PGI_2_ treatment.

## Introduction

Pulmonary arterial hypertension (PAH) is a rare vascular disorder that has an annual incidence of 5 to 8 per million children under the age of 18 (1). With the advances in pharmacological management, the 5-year survival for PAH has risen to 60% over the past decades (2). Continuous intravenous injection of epoprostenol prostaglandin I_2_ (PGI_2_) has been used in patients with severe PAH (2). This medication has contributed to improving the prognosis of primary PAH. However, PGI_2_ may cause a side effect of hyperthyroidism in 6.7% of the subjects (3). Thus, establishing the safest treatment strategies for PAH remains a challenge.

Here we report a 17-year-old boy with congenital porto-systemic venous shunt syndrome (CPSVS), who developed severe hyperthyroidism during PGI_2_ treatment. We also describe the demographic features of previously reported cases with PGI_2_-associated hyperthyroidism by collecting their profiles from the literature.

## Case Report

A 20-day-old male infant was referred to our hospital because of hypergalactosemia detected during neonatal mass screening test. He was diagnosed with congenital portal vein hypoplasia and CPSVS. At seven years of age, PAH was found on regular checkup using echocardiography. Continuous intravenous PGI_2_ (47.2 ng/kg/min) was initiated at nine years of age. The administration of bosentan hydrate (62.5 mg/day) was added at age 10 years. The treatment strategy for his cardiac status was based on World Health Organization (WHO) functional class 2. The right ventricular systolic pressure, estimated from the moderate tricuspid regurgitation, was 80 mmHg on echocardiography. He underwent an assessment of thyroid function once at 16 years of age. The test results showed a low thyroid stimulating hormone (TSH) of 0.04 µU/mL, [reference range (rr): 0.27-4.20] and normal free T4 concentration of 1.42 ng/dL, (rr: 1.00-1.80).

At age 17 years, the patient was admitted to our hospital because of dyspnea, general fatigue and chest pain (WHO class 4). The body temperature was 37.5 ˚C and the heart rate was 120 bpm. On admission, his height was 162.4 cm [-1.1 standard deviation (SD)] and body weight was 44.1 kg (-1.8 SD) resulting in a body mass index of 16.4. Goiter was noted and the liver was palpable at 4.0 cm below the costal margin. Intensified pulmonic sounds with regurgitant systolic murmur was remarkable at the left sternal border. Cardiomegaly was evident on chest radiography. Echocardiography revealed severe tricuspid regurgitation with elevated right ventricular systolic pressure (120 mmHg). A unilateral enlargement of the thyroid gland was detected on ultrasonography with increased blood flow and the estimated thyroid weight was calculated as 3.1 g (right) and 16.7 g (left). Laboratory tests showed a C-reactive protein concentration of 1.8 mg/dL. Brain-type natriuretic peptide was 601.1 pg/mL (cut-off ≤18.4), TSH <0.01 µIU/mL, free T4 at 6.35 ng/dL (rr: 1.00-1.80), thyroid stimulating antibody (TSAb) elevated to 2691% (rr: <180%), TSH receptor antibody (TRAb) level was 10.7 U/L (rr: <1.0 U/L) and thyroglobulin antibody level 1349.7 U/mL (rr: <45 U/L).

**Figure 1:**
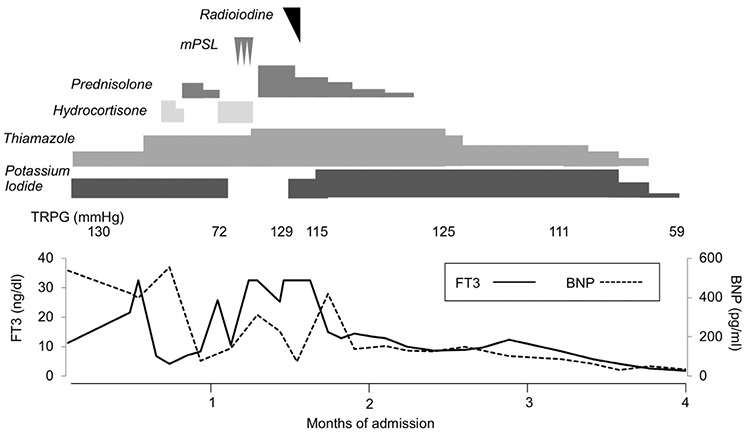
Treatment course of the present case after admission. Applied medications (italics) and their duration of treatment (blocks) are shown at the top. Radioiodine (410 MBq), methylprednisolone (1 g/day for three days), prednisolone (10-45 mg/kg/day), oral administration of thiamazole (15-75 mg/day) and potassium iodide (200-300 mg/day) were used to control the thyrotoxicosis. Echocardiography-based measurements of tricuspid regurgitation peak gradient are shown in the middle. Line charts at the bottom indicate the declining concentrations of free-T3 (reference range: 2.2-4.4 pg/mL) and brain natriuretic peptide (reference range: ≤18.4 pg/mL) over four months of intensive care for the present case mPSL: methylprednisolone, PSL: prednisolone, TRPG: tricuspid regurgitation peak gradient, FT3: free-T3, BNP: brain natriuretic peptide

Maximum doses of oral thiamazole, potassium iodide and intravenous hydrocortisone treatment failed to control the raging storm of hyperthyroidism. High-dose methylprednisolone therapy and destructive radioiodine (RI) (RI in Table 1) therapy were concurrently initiated on the 88th day of admission. Hyperthyroidism gradually improved after the combined therapy. PGI_2_ was continued throughout the period of intensive care because PAH had been severe. When PAH started to improve, the estimated right ventricular pressure declined to 70 mmHg. The patient was discharged 132 days after admission (Figure 1). PAH has been controlled with euthyroid status thereafter. The patient has not received antithyroid therapy for more than four years although TSAb, TRAb and anti-thyroglobulin antibody levels continue to be abnormal. None of his family members were affected by autoimmune thyroiditis. He had no past history of other autoimmune disorders. He had never experienced hypoglycemia, hyperandrogenism or other metabolic attacks before and after this episode.

Written informed consent was obtained from the patient and his parents for the publication of this report.

### Literature Review

We performed a literature search for patients under the age of 20 years who presented with hyperthyroidism during treatment with PGI_2_. We found that 12 such cases had been reported in the years from 2010 to 2017 (4,5). Table 1 summarizes the clinical profiles of these 12 cases and compares with data from our patient (case 13 in Table 1). The median (range) age at diagnosis of PAH was 11 (2-17) years, while the hyperthyroidism developed at a median (range) age of 15.8 (6-19) years. Thus, duration to the development of PGI_2_-associated thyroiditis varied widely from 1 to 11 years after the diagnosis of PAH. Four patients (31%) died of complications including cardiopulmonary dysfunction. We found that six (cases 8-13) among the 13 cases had severe cardiac dysfunction (WHO class 4). Although these six patients underwent thyroidectomy, propylthiouracil or RI therapies, only two (case 12 and the present case) survived the critical period.

## Discussion

We described a case with exacerbated PAH during PGI_2_ treatment. The literature review for the reported cases under 20 years of age indicated a high mortality rate (31%) for PAH patients when complicated by hyperthyroidism. Unfavorable prognosis of PAH was likely to be associated with the severity of cardiac dysfunction at the onset of hyperthyroidism.

PGI_2_ regulates both innate and adaptive immune responses. Recent studies showed evidence that it accelerates the differentiation of naïve T cells into Th17 cells and enhances Th17 cell functions (4,6,7,8). The Th17-interleukin (IL)-17 axis may thus explain the mechanisms of PGI_2_-associated hyperthyroidism and thyroiditis. Considering that the earlier 12 cases presented with hyperthyroidism years after the diagnosis of PAH, the pathogenic mechanisms were less likely to involve acute reactions to PGI_2_. We speculate that deregulation of the physiological immune system by persistent exposure to PGI_2_ in PAH patients might be one of the causes augmenting the pathogenesis of hyperthyroidism. Although we have not analyzed the population of Th17 cells or IL-17 in peripheral blood in our patient, serial immunological studies may detect the prodromal signs of hyperthyroidism in PAH patients.

Experimental studies demonstrated that PGI_2_ regulates both innate and adaptive immune systems (9). PGI_2_ analogs were also shown to inhibit proinflammatory responses to lipopolysaccharides in monocyte and macrophage populations (10). Notably, inflammatory macrophage populations were reported to be expanded in the lungs a mouse model of PAH (11). Thus, delineating the downstream signals to PGI_2_ in the lung macrophage will be the key to understand its deleterious effects on thyroid functions. Among them, monocyte chemoattractant protein-1 (MCP-1/CCL2) is known as a downstream molecule following prostaglandin stimulation (12). Paradoxical effects of PGI_2_ on thyroid functions might therefore result from differential MCP-1 synthesis in each tissue as a result of long-term treatments.

We considered that the exacerbation of PAH was a consequence not only of the increased cardiac outputs with hyperthyroidism, but also from the direct effect of thyroid hormone on proliferative vascular endothelial cells (13). Together with our case report, the literature review also supports the necessity of prophylactic monitoring and management of thyroid function for PAH patients undergoing PGI_2_ treatment. Earlier intervention may prevent PAH patients from the progressive worsening of cardiac dysfunction. In this regard, prophylactic therapy might have been helpful if initiated in our patient at age 16 years, when he showed a low TSH concentration on thyroid testing. Future studies will clarify whether this alternative strategy might have changed the unfavorable outcomes of these patients.

## Figures and Tables

**Table 1:**
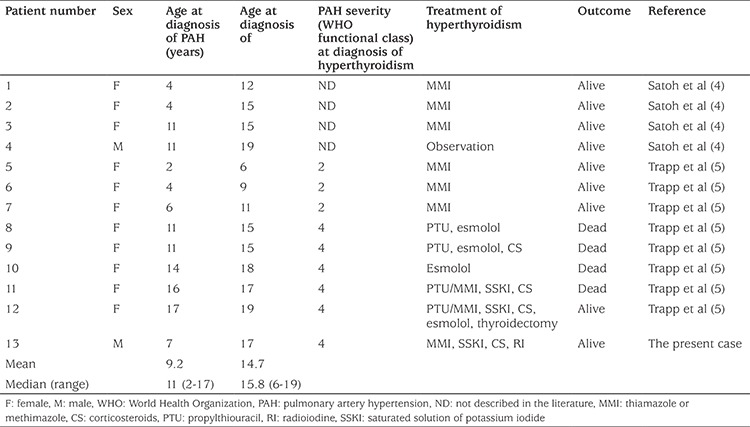
Clinical characteristics of pediatric pulmonary artery hypertension patients complicated with hyperthyroidism during PGI_2_ treatment
